# Stimulated Raman scattering microscopy with spectral phasor analysis: applications in assessing drug–cell interactions†

**DOI:** 10.1039/d1sc06976d

**Published:** 2022-02-25

**Authors:** William J. Tipping, Liam T. Wilson, Connie An, Aristea A. Leventi, Alastair W. Wark, Corinna Wetherill, Nicholas C. O. Tomkinson, Karen Faulds, Duncan Graham

**Affiliations:** Centre for Molecular Nanometrology, WestCHEM, Department of Pure and Applied Chemistry, Technology and Innovation Centre, University of Strathclyde Glasgow G1 1RD UK karen.faulds@strath.ac.uk duncan.graham@strath.ac.uk; Department of Pure and Applied Chemistry, University of Strathclyde Glasgow G1 1XL UK nicholas.tomkinson@strath.ac.uk

## Abstract

Statins have displayed significant, although heterogeneous, anti-tumour activity in breast cancer disease progression and recurrence. They offer promise as a class of drugs, normally used for cardiovascular disease control, that could have a significant impact on the treatment of cancer. Understanding their mode of action and accurately assessing their efficacy on live cancer cells is an important and significant challenge. Stimulated Raman scattering (SRS) microscopy is a powerful, label-free imaging technique that can rapidly characterise the biochemical responses of live cell populations following drug treatment. Here, we demonstrate multi-wavelength SRS imaging together with spectral phasor analysis to characterise a panel of breast cancer cell lines (MCF-7, SK-BR-3 and MDA-MB-231 cells) treated with two clinically relevant statins, atorvastatin and rosuvastatin. Label-free SRS imaging within the high wavenumber region of the Raman spectrum (2800–3050 cm^−1^) revealed the lipid droplet distribution throughout populations of live breast cancer cells using biocompatible imaging conditions. A spectral phasor analysis of the hyperspectral dataset enables rapid differentiation of discrete cellular compartments based on their intrinsic SRS characteristics. Applying the spectral phasor method to studying statin treated cells identified a lipid accumulating phenotype in cell populations which displayed the lowest sensitivity to statin treatment, whilst a weaker lipid accumulating phenotype was associated with a potent reduction in cell viability. This study provides an insight into potential resistance mechanisms of specific cancer cells towards treatment with statins. Label-free SRS imaging provides a novel and innovative technique for phenotypic assessment of drug-induced effects across different cellular populations and enables effective analysis of drug–cell interactions at the subcellular scale.

## Abstract 2

Stimulated Raman scattering microscopy with spectral phasor analysis provides a label-free approach for phenotypic evaluation of drug-induced effects.

## Graphical abstract

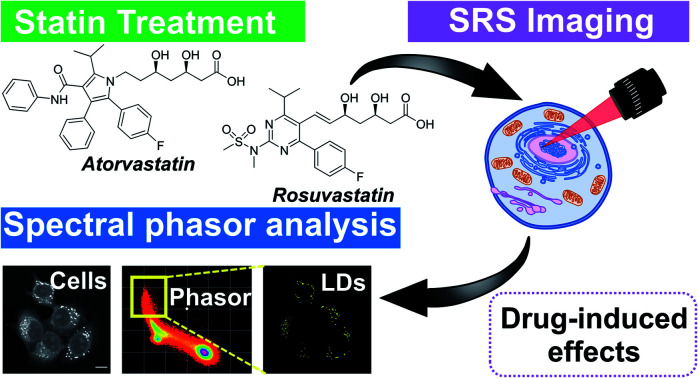

## Introduction

Statins are potent inhibitors of 3-hydroxy-3-methylglutryl coenzyme A (HMGC) reductase, a key enzyme in the mevalonate pathway that ultimately results in cholesterol biosynthesis. Statins are routinely prescribed to lower serum cholesterol levels and thus decrease cardiac morbidity and mortality.^1^ In addition, consistent evidence supports a protective effect of several statins in breast cancer disease recurrence.^2^ Putative anticancer mechanisms include inhibition of proliferation by global cholesterol reduction and inhibition of oncogenic signaling, among others.^3^ However, in both preclinical models and breast cancer patients, the response to various statin treatments has been shown to be heterogeneous.^4^ For example, in murine models of breast cancer, atorvastatin has been shown to suppress proliferation at metastatic sites but not within the primary tumour.^5^ As such, effective methods for the identification of statin-induced effects on lipid metabolism and regulation in cancer cells are required.^6^

Previous studies have used brightfield^7^ and fluorescent microscopy^8^ to visualise intracellular lipid dynamics associated with statin treatment in breast and pancreatic cancer cells, respectively. The hydrophobic dyes, Oil Red O and Nile Red, used for contrast in these techniques, intrinsically perturb the lipid droplet composition and dynamics with confounding negative implications.^9^ For example, hydrophobic dyes disrupt the biophysical properties of the lipid droplet membrane and the composition of the droplet, directly impacting intracellular motility and localisation. In a comparative study, transmission electron microscopy (TEM) identified a lipid increase in osteosarcoma cells treated with simvastatin;^10^ however, this imaging method is not compatible with living systems. Label-free detection methods of lipid dynamics in living cells would provide a clear advantage to the currently reported approaches.

Raman spectroscopy is a preferred method for biochemical analysis because it offers label-free, non-destructive detection of cellular biomolecules with chemical specificity under biocompatible conditions. Notably, it has been used previously to study lipid droplets^11^ and metabolic alterations during trametinib treatment^12^ in fixed breast cancer cells, although the image acquisition rates and spatial resolution of Raman spectroscopy can restrict the throughput of the technique.

Coherent Raman imaging techniques have brought about improvements in three-dimensional (3D) imaging capability, spatial resolution, and temporal analysis.^13^ In particular, stimulated Raman scattering (SRS) microscopy has been applied to the detection of protein, lipid and DNA dynamics in living cells, tissues and animal models.^14,15^ In addition, SRS is becoming established as a robust imaging modality in drug discovery;^16^ notably for drug distribution studies of tyrosine kinase inhibitors,^17,18^ natural products^19,20^ and agrochemical agents.^21^ A key strength of SRS imaging compared to Raman scattering is the fast image acquisition rate, which can enable real-time imaging of biological processes in living systems.^22^ Furthermore, hyperspectral SRS microscopy can be performed for reliable sample characterisation based on the SRS spectrum. By applying spectral phasor analysis directly to hyperspectral SRS images, Fu and Xie demonstrated reproducible identification of cellular organelles based directly on their respective SRS spectra.^23^ Spectral phasor analysis projects the SRS spectrum from each pixel within the hyperspectral stack onto a two-dimensional phasor domain, where pixels with similar SRS spectral features are clustered together. This methodology has recently enabled SRS-based cytometery,^24^ although it has yet to be applied to studying drug–cell interactions, which has accordant potential in the drug development process.

Herein, we describe the application of SRS microscopy combined with a spectral phasor analysis for label-free characterisation of breast cancer cells exposed to increasing concentrations of two clinically relevant statins, atorvastatin and rosuvastatin. We applied a spectral phasor analysis of hyperspectral SRS image stacks that enabled a robust and reproducible means to localise intracellular lipid compartments with chemical specificity. Our results provided a label-free insight into the association of statin treatment on lipid dynamics in breast cancer cells, and enabled the first, direct comparison between hydrophobic and hydrophilic statins in this area. Our results demonstrate that SRS microscopy is a compelling method for use in phenotypic assessment of drug-induced effects on cellular lipid dynamics in cells.

## Results and discussion

**Fig. 1:**
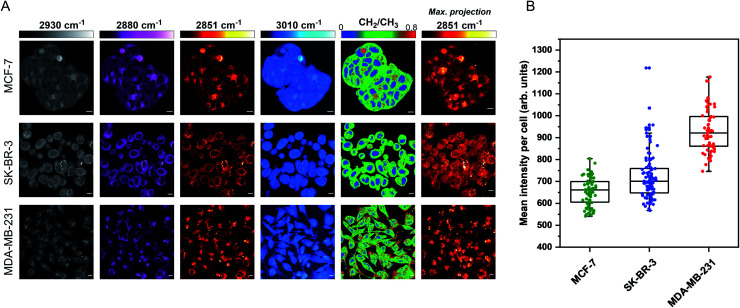
Characterisation of live breast cancer cells using stimulated Raman scattering microscopy. (A) MCF-7, SK-BR-3 and MDA-MB-231 cells were imaged using SRS microscopy at the following frequencies: 2930 cm^−1^ (CH_3_ symmetric stretch), 2880 cm^−1^ (CH_2_ asymmetric stretch), 2851 cm^−1^ (CH_2_ symmetric stretch) and 3010 cm^−1^ (=CH stretch). A ratiometric image of the CH_2_/CH_3_ (2851 cm^−1^/2930 cm^−1^) is also presented. The background (non-cell areas) has been removed using an intensity threshold (see Experimental for details). A maximum intensity projection from a Z-stack of images acquired at 2851 cm^−1^ (from the same cellular population) is also provided. Single-frequency SRS images were acquired at a frame size of 1024 × 1024 pixels, using a 24 μs pixel dwell time with false colours applied to different detection wavenumbers. Scale bars: 10 μm. (B) Mean intensity of 2851 cm^−1^ (CH_2_) signal per cell quantified in >50 cells from the maximum intensity projection presented in (A).

The aim for this research was to investigate if statin treatment would trigger a varying lipid profile as a consequence of altered lipid metabolism. To that end, it would provide a novel insight into the potential treatment of breast cancer using statins. To investigate the effects of statin treatment in breast cancer cells, we identified three cell lines with different subtypes and expression profiles of estrogen receptor-α (ERα) (Fig. S1†): MCF-7 (luminal, ERα+), SK-BR-3 (luminal, ERα−) and MDA-MB-231 (basal B, ERα−).^25^ ERα expression has previously been linked to therapy resistance mechanisms in breast cancer cells.^4^ We employed label-free SRS imaging to characterise the global protein and lipid distributions in the three selected breast cancer cell lines (Fig. 1A). SRS images were acquired by tuning the frequency difference between the pump beam and Stokes beam to be resonant with intracellular proteins (2930 cm^−1^, CH_3_ symmetric stretch) and lipids (2880 cm^−1^ & 2851 cm^−1^, CH_2_ asymmetric and symmetric stretch, and 3010 cm^−1^, =CH lipids).^26^ The images acquired at 2930 cm^−1^ highlight protein and lipid signal throughout the cell cytoplasm, cell nuclei and nucleoli in each of the three cell lines. Notably, MCF-7 cells display an endothelial morphology with strong cell-to-cell adhesion, whilst MDA-MB-231 cells displayed a characteristic elongated form.^27^ In addition, lipid droplets were detected in all three cell lines in the images acquired at 2851 cm^−1^.^28^ Ratiometric images were generated from CH_2_/CH_3_ which resolved intracellular features including nuclear regions with a reduced CH_2_ ratio, whilst lipid droplets were detected in the cytoplasm in each of the three cell lines, and were most abundant in the MDA-MB-231 cells. Notably, almost all MDA-MB-231 cells contained lipid droplets (Fig. S2†), whilst a decreasing trend in the percentage of cells containing lipid droplets for SK-BR-3 cells (∼40%) and MCF-7 cells (∼20%) was observed.

Three-dimensional (3D) image Z-stacks were acquired across each cell population at 2851 cm^−1^ by adjusting the focal plane (*Z* = 1 μm) between image frames. A maximum intensity projection§§A maximum intensity projection creates an output image from a Z-stack series, where each of the pixels in the projection contains the maximum value over all images in the stack at each particular pixel location. was then created from the image Z-stack, which rendered the 3D dataset into a 2D image based on the maximum voxel intensity (Fig. 1A). Using this approach, it was possible to assess the lipid distribution throughout the entire volume of the cell population. The intensity of the lipid signal across three replicate images from each cell line was quantified and the data presented in Fig. 1B. MDA-MB-231 cells showed a higher 2851 cm^−1^ signal than SK-BR-3 and MCF-7 cells, respectively. This observation is in agreement with the analysis of the percentage of breast cancer cells in each population containing lipid droplets (presented in Fig. S2†). Finally, off-resonance images were acquired at 2800 cm^−1^ (Fig. S3†), where no cellular Raman bands are expected; these images confirmed the minimal non-resonant background contribution associated with SRS imaging.^29^

**Fig. 2:**
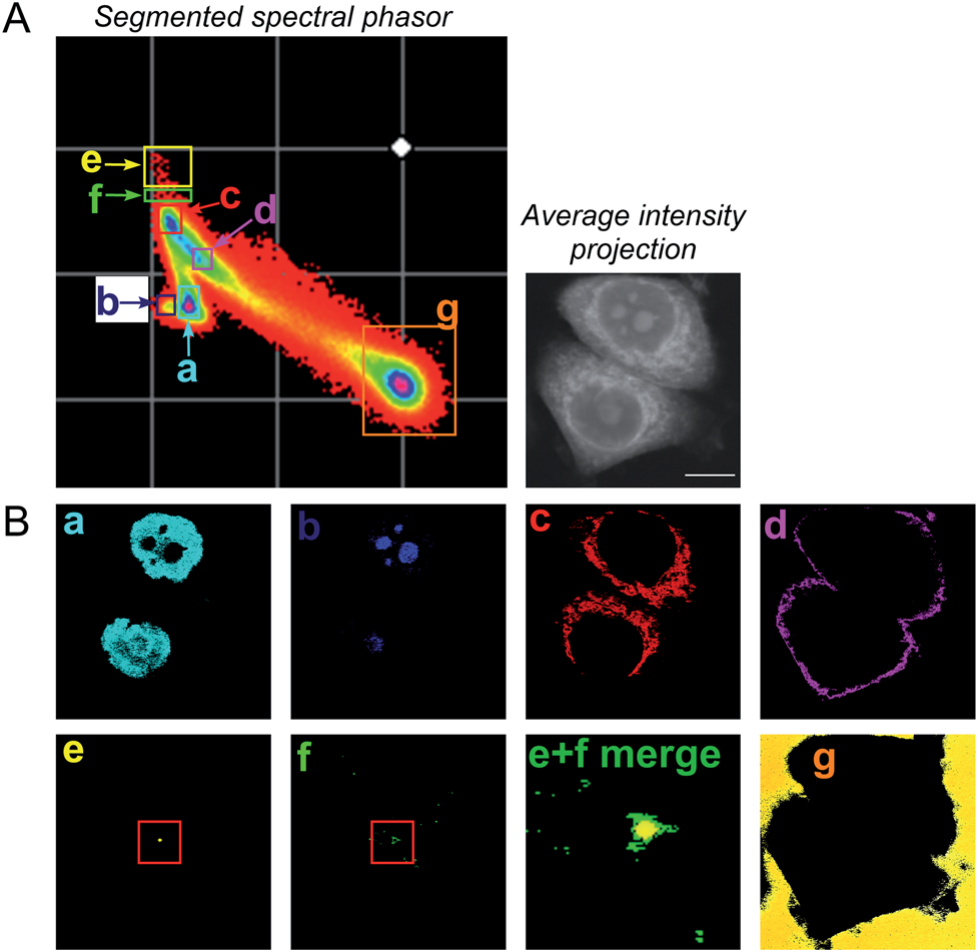
A spectral phasor analysis of SRS images acquired from fixed SK-BR-3 cells. (A) SRS images were acquired across the range 2800–3050 cm^−1^ and a spectral phasor analysis performed on the image stack. The spectral phasor plot has been segmented into 7 regions of interest (ROIs) as indicated by the coloured boxes and corresponding labels (a–g). An average intensity projection of the hyperspectral stack is also provided (scale bar: 10 μm). (B) Corresponding segmented images from the ROIs highlighted in the spectral phasor plot. The segmented images are colour-matched to the ROI markers in (A). In (e) (lipid droplet) and (f) (lipid droplet periphery), a red marker has been added to show the region expanded in the merged image (labelled (e) + (f) merge).

To improve the accuracy associated with identifying lipid-rich regions within the cell population, we elected to apply a spectral phasor approach as described by Fu *et al.*^23^ To do so, we performed a wavelength scanning experiment, whereby the pump laser wavelength was retuned in increments of 0.4 nm (∼6 cm^−1^, 40 images), with subsequent acquisition of an SRS image at each wavenumber to create a hyperspectral SRS image stack across the range 2800–3050 cm^−1^ (a representative example is provided in Fig. S4†). The three-dimensional data stack was transformed into a two-dimensional phasor plot based on a Fourier transform as described previously by Fu *et al.*^23^ The raw SRS spectral data set was imported directly into the spectral phasor analysis without any post-processing or spectral normalisation. Each data point (referred to as a spectral phasor) on the phasor plot represents a voxel within the 3D stack, and therefore corresponds to a unique SRS spectrum within the stack. The spatial closeness of any two phasors is determined by the SRS spectral similarity of the pixels that they represent. Thus, a cluster of spectral phasors indicates pixels that share similar SRS spectra, which can then be mapped back to an area in the sample image. Therefore, the phasor plot can be segmented based on these clusters to identify distinct intracellular features based directly upon the SRS spectra associated with them. For example, SK-BR-3 cells were segmented into 7 regions of interest, representing: (a) the nucleus, (b) nucleoli, (c) cytoplasm, (d) cell periphery, (e) lipid droplets, (f) lipid droplet periphery, and (g) background/non-cell areas based on similar features identified by Fu *et al.* (Fig. 2).^23^

**Fig. 3:**
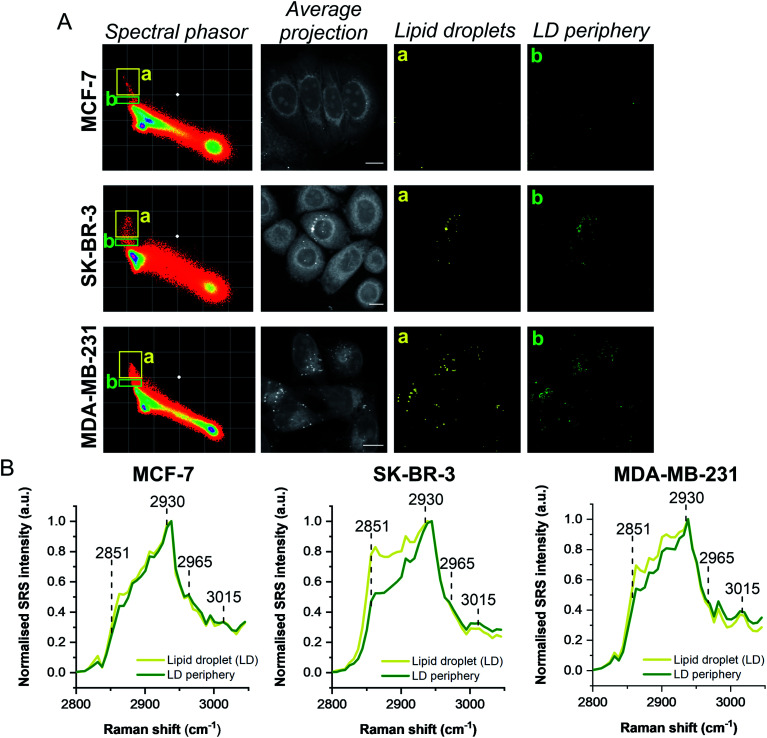
Comparison of breast cancer cell lines using a spectral phasor approach. (A) Hyperspectral SRS images were acquired from populations of MCF-7, SK-BR-3 and MDA-MB-231 cells. Spectral phasor plots were created for each SRS image stack together with an average intensity projection (scale bar: 10 μm). Colour-coded images of the lipid-rich region of the spectral phasor plot were constructed based on the ROIs identified by the yellow (a, lipid droplets, LDs) and green ROIs (b, LD periphery), respectively. (B) Normalised average SRS spectra for the ROIs identified in (A). The spectra are normalised between 0–1.

A schematic workflow detailing the data acquisition and phasor analysis is provided in Fig. S5.† The colour-coded segments of the phasor plot enabled reliable delineation of the hyperspectral image stack into a 2D representation of these key regions. Of particular interest, was the ability to differentiate lipid droplets (e, yellow) and the surrounding periphery (f, green) from the rest of the cell cytoplasm, consequently improving the accuracy in the measurement of lipid content within the cells. As such, we investigated the lipid content of MCF-7, SK-BR-3 and MDA-MB-231 cells (Fig. 3A). To simplify the analysis, we focused on the region of the phasor plot associated with lipid droplets as identified by the yellow ROI (e). The region in the spectral phasor plot highlighted by the green ROI (f), is associated with the periphery of the lipid droplets. The average SRS spectra of the pixels within each of these regions is plotted in Fig. 3B. From these spectra, an increased ratio of the 2851 cm^−1^/2930 cm^−1^ (CH_2_/CH_3_) was associated with the lipid droplet (yellow) when compared to the surrounding lipid droplet periphery regions (green) which have a higher protein (2930 cm^−1^) content. When comparing the spectral phasor plots and the associated segmented images for the three cell lines, an increase in lipid content is detected from MCF-7 to SK-BR-3 and MDA-MB-231 cells. Analysis of the yellow ROIs in the phasor plot for SK-BR-3 and MDA-MB-231 cells, also show an increasing number of phasors (and hence voxels in each image stack) that have a lipid-rich spectrum when compared to the MCF-7 cells. This observation is consistent with the analysis presented in Fig. 1. As such, phasor analysis of SRS spectral data sets represents a rapid and reliable means to study the composition of different lipid regions across a range of cell lines. In addition, as a label-free technique, our analysis is achieved without impacting the composition or perturbing the biophysics of the lipid droplets which is a limitation of hydrophobic fluorescent stains.

**Fig. 4:**
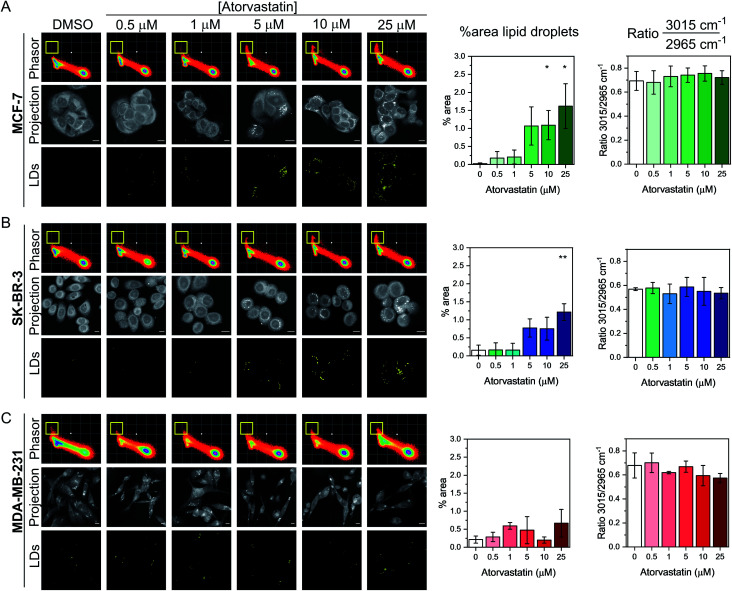
Investigating the effect of atorvastatin in breast cancer cells using a spectral phasor approach. Spectral phasor plots were generated from (A) MCF-7, (B) SK-BR-3 and (C) MDA-MB-231 cells treated with atorvastatin at the indicated concentrations for 48 h. An average intensity projection from the SRS spectral sweep is presented alongside the segmented phasor plot indicating lipid droplets (LDs, identified by the yellow ROI in the spectral phasor plot). Scale bars: 10 μm. For each cell line, the percentage area of lipid droplets in the segmented image is expressed as a function total cell area (determined from the average intensity projection), and the ratio of the intensity of the peaks at 3015 cm^−1^/2965 cm^−1^ is plotted as a function of atorvastatin concentration. Three replicate analyses were obtained from >30 cells for each condition. Data represent the mean ratio ± S.D. A Student's *t*-test is performed on the percentage area data, *P ≤ 0.05, **P ≤ 0.01.

Having demonstrated the utility of the phasor analysis for reliable cell segmentation, we next studied the effect of statin treatment in each cell line. We elected to study the effects of atorvastatin (log *P* = 4.1) and rosuvastatin (log *P* = −0.3) which are classified as hydrophobic and hydrophilic statins, respectively (Fig. S6†).^30^ The cells were first treated with varying doses of either statin (0.5–25 μM) for 48 h, whilst control cells were concomitantly treated with DMSO. SRS images were acquired across the range 2800–3050 cm^−1^ (40 images) and an average intensity projection from the 3D stack was generated as a label-free means to localise the cells in each imaging experiment. Spectral phasors were then created for each population of cells for each treatment condition. The data for the atorvastatin treatments are presented in Fig. 4, whilst the rosuvastatin treatments are presented in Fig. S7.† A yellow ROI was added to each phasor plot to identify the lipid droplet regions, from which the segmented image of lipid droplets (LDs) was prepared. As can be clearly observed in the phasor plots and the segmented images of the MCF-7 cells, there was an increase in lipids at atorvastatin treatment concentrations >5 μM (Fig. 4A). We quantified the % area of lipid droplets in the segmented image as a function of total cell area (determined from the average intensity projection) which confirmed significant lipid droplet accumulation at the higher atorvastatin concentrations. To highlight the capability of the spectral phasor analysis for delineating regions of interest in the cell populations (as per Fig. 2), we present the segmentation of MCF-7 cells treated with DMSO (control) and atorvastatin (5 μM, 48 h) as representative examples in Fig. S8.†

The spectral phasor analysis of SK-BR-3 cells also showed an increase in lipid droplets at atorvastatin concentrations >10 μM (Fig. 4B), whilst in the case of MDA-MB-231 cells, there was no apparent variation in the lipid content of the cells across the range of atorvastatin concentrations tested (Fig. 4C). Furthermore, for each cell line, we assessed the ratio of the intensity at 3015 cm^−1^/2965 cm^−1^ which has been shown previously to act as an indicator for assessing the composition of lipid droplets comprising triacylglycerols (TAGs, 3015 cm^−1^, =CH) and/or cholesterol esters (CEs, 2965 cm^−1^).^31^ Across the three cell lines, we observed no significant variation in this key ratio value at all atorvastatin concentrations tested. Indeed, the 3015 cm^−1^/2965 cm^−1^ ratio values were consistently ∼0.7, which corroborated a previous analysis that showed the ratio 3015 cm^−1^/2965 cm^−1^ of lipid droplets formed predominantly of TAGs was 0.75.^31^ This observation is surprising given that statins are known inhibitors of cholesterol biosynthesis through inhibition of HMGC reductase, and in each of the three cell lines analysed in this work, the CE content associated with lipid droplets in both the control and atorvastatin treated cells appeared to be low.

To validate our findings using SRS microscopy and spectral phasor analysis, we performed spontaneous Raman spectroscopy on the panel of breast cancer cells exposed to DMSO (control) or atorvastatin (1 μM or 10 μM). The average cellular Raman spectra in the fingerprint region (400–2000 cm^−1^) and high wavenumber region (2800–3100 cm^−1^) are presented in Fig. S9A–C.† Increased lipid content was observed in the average Raman spectra acquired in MCF-7 and SK-BR-3 cells at the concentrations tested particularly at 1446 cm^−1^ (CH_2_ bending) and 2851 cm^−1^ (CH_2_ symmetric stretch), whilst a weaker lipid accumulation is observed at these frequencies in the MDA-MB-231 cells. Furthermore, we noted that the Raman spectra of lipid droplets in MCF-7 cells presented intense peaks indicative of fatty acids (Fig. S9D†), with the notable observation that there was negligible evidence of peaks at 701 cm^−1^ (cholesterol ring vibration) and 1670 cm^−1^ (sterol C=C bond) indicative of CEs.^32^ This finding indicates that the accumulated lipid droplets in MCF-7 cells generally presented low cholesterol content in agreement with our SRS and spectral phasor analysis (Fig. 4).

Lastly, we investigated the effect of rosuvastatin on the three breast cancer cell lines using spectral phasor analysis (Fig. S7†). Rosuvastatin is a hydrophilic statin, and a weak lipid accumulating phenotype was observed in MCF-7 and SK-BR-3 cells at concentrations >10 μM. It is also worthy of mention that negligible effects in the lipid region of the spectral phasor plot were observed in MDA-MB-231 cells, which is in concurrence with the effects observed with atorvastatin. The general observation in all cell lines, was that atorvastatin treatment produced a more pronounced effect on lipid metabolism than rosuvastatin did. The data presented here also suggested that ERα+ MCF-7 cells have a greater potential to accumulate lipid droplets upon statin exposure when compared to the corresponding ERα− MDA-MB-231 cells, but not ERα− SK-BR-3 cells. These data therefore suggest that the lipid accumulation phenotype is independent of ERα status.

**Fig. 5:**
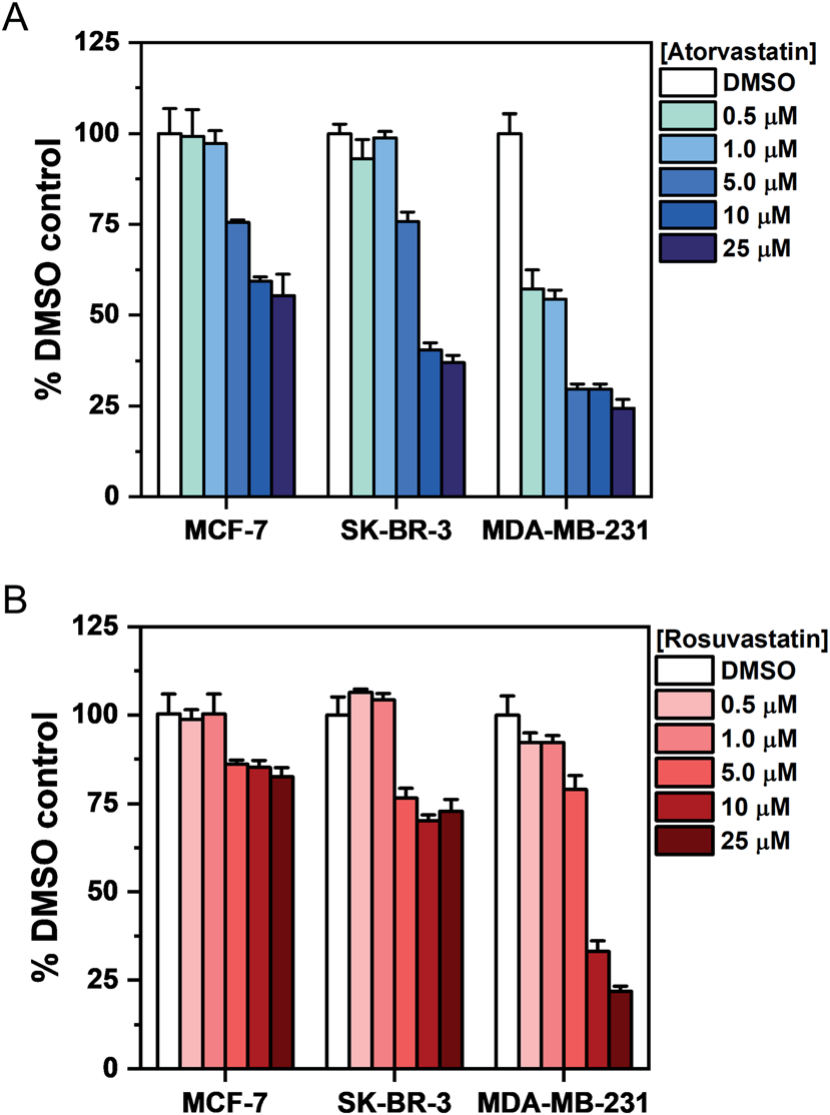
Assessment of breast cancer cell viability upon exposure to atorvastatin and rosuvastatin using Trypan blue staining. Cells were exposed to DMSO (control) or an increasing concentration of either (A) atorvastatin or (B) rosuvastatin for 48 h. The number of viable cells was determined using Trypan blue staining and expressed as a mean% of the DMSO control with error bars: +S.D.

To investigate the association between statin treatment, lipid accumulation and cell toxicity, we performed a Trypan blue cell viability assay for each treatment condition (Fig. 5). Interestingly, atorvastatin exerted a greater toxic response in all three cell lines when compared to rosuvastatin. In addition, sensitivity of statin treatment was greatest in MDA-MB-231 cells, whereas SK-BR-3 cells presented moderate sensitivity and MCF-7 cells were identified as least sensitive to statin treatment. These data indicate that resilience to statin treatment is correlated with an increased lipid accumulation in MCF-7 cells; whilst MDA-MB-231 cells, which have higher basal levels of lipid droplets and showed greatest sensitivity to statin treatment, produced only marginal changes in lipid accumulation following treatment. Thus, resistance to statin treatment appears to correlate positively with increased lipid accumulation in breast cancer cells. In addition, our results generally agree with previous reports documenting the impact of gene expression and sensitivity to statin treatment.^4^ Specifically, Kimbung *et al.* showed that cell lines which presented least sensitivity to statin treatment (including MCF-7 cells) were capable of strongly inducing the expression of genes involved in the cholesterol biosynthesis pathway because of statin-induced inhibition of HMGC reductase.^4^ Meanwhile, a weaker gene expression profile was observed in statin-sensitive cells, which was associated with an impairment in cell viability.

Given the association between statin sensitivity and lipid droplet accumulation, we reasoned it would be fruitful to determine if *de novo* lipid synthesis or exogenous lipid uptake from culture medium was the likely origin of the intracellular lipid content. We therefore cultured MCF-7 cells with atorvastatin or rosuvastatin under serum-free conditions (Fig. S10†). Our results indicated that in serum-free media, cells which were treated with increasing atorvastatin concentrations resulted in lipid accumulation, particularly at higher (>5 μM) concentrations (Fig. S10A†), whilst a weaker lipid accumulation effect was detected in the rosuvastatin treated cells (Fig. S10B†). The impact on cell viability of each statin in serum-free media was also assessed and produced comparable results to the viability study in complete media (Fig. 5, S10C and D†). Together, these results indicate that the lipid accumulation in MCF-7 cells likely arises from *de novo* lipid synthesis.

Lipid droplets have been associated with all processes involved in cancer development, including initiation, promotion and progression.^33^ Statins have been shown to result in a protective effect in breast cancer recurrence,^34^ and reduce the risk of breast cancer related mortality.^35^ The mechanism by which anticancer properties of statins are exerted is as yet unknown. The results described here indicate that upon treatment with atorvastatin or rosuvastatin, an increase in cellular lipid content is observed in MCF-7 cells which displayed the greatest resilience (least toxicity) to the treatment. SK-BR-3 cells produced similar results to the MCF-7 cells, which suggests that statin sensitivity is independent of ERα expression. Additionally, MDA-MB-231 cells conferred a reduced level of lipid droplet accumulation, whilst an increased toxic response was determined when compared to the MCF-7 cells. It is interesting to note that resistance to trametinib, an inhibitor of the extracellular-signal-regulated kinase (ERK) pathway, was observed in MCF-7 cells whilst MDA-MB-231 were shown to be sensitive to this drug treatment.^12^ The lipid accumulating phenotype observed here builds on previously observed lipid increases in keratinocytes^36^ and a range of cancer cell lines including breast,^7^ pancreatic,^8^ and bone cancer^10^ cells upon exposure to a variety of different statins. The distinct advantage of the SRS imaging approach described here is that the characterisation of intracellular lipid droplets was determined in cells without the requirement for hydrophobic dyes for chemical contrast. The spectral phasor analysis also enabled a reliable and reproducible means to localise lipid droplets with chemical specificity based on the SRS spectral profile.

Our results also indicated that atorvastatin treatment generally produced a stronger lipid accumulating phenotype in MCF-7 cells and greater effects on cell viability (in all cell lines) than corresponding treatments with rosuvastatin. This observation is in spite of the fact that rosuvastatin has a higher affinity for HMGC reductase than atorvastatin.^37^ A potential explanation for this observation may be increased uptake of hydrophobic statins, including atorvastatin, *via* passive diffusion when compared to hydrophilic statins including rosuvastatin. Ahern *et al.* hypothesised that hydrophobic statins have the greatest potential to exert additional effects beyond lowering serum cholesterol levels,^3^ and our results generally agree with this hypothesis. Our report is the first to correlate specific cellular lipid metabolism with hydrophobic or hydrophilic statins.

## Conclusions

We reported the use of SRS microscopy for assessing the phenotypic outcome of atorvastatin and rosuvastatin treatment on cellular lipid metabolism in a panel of breast cancer cell lines. Label-free imaging of intracellular lipid content in live cells represents a distinct advantage to currently reported imaging modalities, that require either the use of fluorescent labels for contrast which intrinsically perturb the droplet composition and biophysics, or are destructive, as is the case for TEM or mass spectrometry-based imaging techniques. Phasor analysis provides a graphical way of globally visualizing the spectral variations of a cell as a function of its composition. In this case, the intracellular lipid content is directly observed in the phasor plots of all cell lines tested, enabling straightforward analysis of drug-induced effects on total lipid content. Our results indicated a significant increase in lipid droplets in MCF-7 cells, which also showed a concomitant resistance to statin treatment. Conversely, SK-BR-3 and MDA-MB-231 cells gave a reduced lipid accumulation phenotype whilst presenting a greater impact on cell viability following statin treatment. These data indicate a link between increased lipid metabolism as a potential resistance and survival mechanism in breast cancer cells. To that end, phenotypic evaluation of drug-induced effects using SRS microscopy has enabled a unique insight into studying drug activity at the single-cell level.

## Experimental

### Reagents and chemicals

Atorvastatin calcium trihydrate (Sigma Aldrich) and rosuvastatin calcium (Thermo Fisher Scientific) were used as supplied and prepared as 50 mM stock solutions in anhydrous DMSO.

### Cell culture

MCF-7 cells (ATCC® HTB-22™) and SK-BR-3 cells (ATCC® HTB-30™) were obtained from American Type Culture Collection (ATCC). MDA-MB-231 cells (ATCC® HTB-26™) were gifted from the Strathclyde Institute of Pharmacy and Biomedical Sciences (Glasgow) as a subculture from a stock received from the European Collection of Authenticated Cell Cultures (ECACC). All breast cancer cell lines were cultured in Rosewell Park Memorial Institute medium (RPMI 1640; GIBCO™, Fisher Scientific) supplemented with 10% foetal bovine serum (FBS, Gibco™, Fisher Scientific), 1% penicillin/streptomycin (Gibco™, 10 000 U mL^−1^, Fisher Scientific) and 1% amphotericin B (Gibco™, 250 μg mL^−1^, Fisher Scientific). Cells were maintained at 37 °C and 5% CO_2_ in a humidified incubator and were routinely sub-cultured at *ca.* 80% confluency.

### SRS microscopy

An integrated laser system (picoEmerald™ S, Applied Physics & Electronics, Inc.) was used to produce two synchronised laser beams at 80 MHz repetition rate. A fundamental Stokes beam (1031.4 nm, 2 ps pulse width) was intensity modulated by an electro-optic-modulator (EoM) with >90% modulation depth, and a tunable pump beam (700–960 nm, 2 ps pulse width, <1 nm (10 cm^−1^) spectral bandwidth) was produced by a built-in optical parametric oscillator. The pump and Stokes beams were spatially and temporally overlapped using two dichroic mirrors and a delay stage inside the laser system and coupled into an inverted laser-scanning microscope (Leica TCS SP8, Leica Microsystems) with optimised near-IR throughput. SRS images were acquired using 40× objective (HC PL IRAPO 40×, N.A. 1.10 water immersion lens) with a 9.75–48 μs pixel dwell time over a 512 × 512 or a 1024 × 1024 frame. The Stokes beam was modulated with a 20 MHz EoM. Forward scattered light was collected by a S1 N. A. 1.4 condenser lens (Leica Microsystems). Images were acquired at 12-bit image depth. The laser powers measured after the objective lens were in the range 10–30 mW for the pump beam only, 10–50 mW for the Stokes beam only and 20–70 mW (pump and Stokes beams). The spatial resolution of the system is ∼450 nm (pump wavelength = 792 nm).

#### SRS imaging and spectral phasor analysis

MCF-7, SK-BR-3 and MDA-MB-231 cells were plated on high precision glass coverslips (#1.5H thickness, 22 × 22 mm, Thorlabs) in a 6-well plate in RPMI at a concentration of 5 × 10^5^ cells per mL and incubated at 37 °C and 5% CO_2_ for a 24 h prior to treatment. Cells were treated with atorvastatin or rosuvastatin from a 50 mM stock solution in DMSO (or DMSO as a control) and incubated at 37 °C and 5% CO_2_ for the indicated time. Prior to imaging, the plates were aspirated and washed with PBS (2 × 2 mL), the cells were fixed with paraformaldehyde (4% in PBS, 15 min at rt), and washed with PBS (2 × 2 mL). The coverslips were then affixed to glass microscope slides with a PBS boundary between the glass layers prior to imaging following the method described in ref. 17. Z-stacks were acquired at 1 μm increments in the *Z* plane. For live cell imaging, the cells were washed with PBS (2 × 2 mL) following the relevant treatment, before mounting onto glass microscope sides as described.

### Cell viability assessment

#### Trypan blue assay

MCF-7, SK-BR-3 and MDA-MB-231 cells were seeded onto a 6-well plate in RPMI at a concentration of 5 × 10^5^ cells per mL and incubated at 37 °C and 5% CO_2_ for 24 h prior to treatment. The cells were washed with PBS (2 mL) before treatment with either DMSO (0.025% v/v negative control), atorvastatin (0.5–25 μM) or rosuvastatin (0.5–25 μM) in RPMI for 48 h. After 48 h, the media was removed, the cells rinsed with PBS (2 × 2 mL) before the addition of 0.25% trypsin–EDTA (500 μL) to detach the cells at 37 °C. The trypsin was deactivated by the addition of RPMI media (500 μL) and the cells were collected by gentle aspiration with a pipette. The cell suspension (10 μL) was diluted with 0.4% Trypan blue (10 μL) and live cells counted using a haemocytometer. The number of live cells for each condition is expressed as a % relative to the DMSO control.

### Data processing

#### SRS images

False colour assignments, scale bars and image overlays were added to images using ImageJ software. Consistent brightness and contrast settings were used when comparing image datasets. For the lipid droplet analysis, a Z-stack of SRS images at 2851 cm^−1^ (CH_2_, lipids) was acquired across a typical field of view for each treatment condition and a maximum intensity projection generated using ImageJ. The mean SRS intensity per cell was determined by first, manually selecting individual cells using the freehand selection tool, and the intensity determined using ImageJ analysis tool. For CH_2_/CH_3_ ratio imaging, a threshold (mask) image was first generated by adjusting threshold on ImageJ, then non-zero values were normalized to one. CH_2_ images were then divided by the corresponding CH_3_ image, and the resulting ratio image multiplied with the mask image to create the final CH_2_/CH_3_ ratio image. The display range of CH_2_/CH_3_ ratio images is set to be 0–0.8 and are presented in the Rainbow RGB LUT.

#### Spectral phasor analysis

The SRS image data set across the range 2800–3050 cm^−1^ was imported into ImageJ and an average intensity projection was created. The spectral phasor analysis was performed as described by Fu *et al.*^23^ using a plug-in for ImageJ that is available online (http://www.spechron.com/Spectral-Phasor-Download.aspx, accessed 10^th^ January 2022). Segmentation of the phasor plot was performed manually using regions-of-interest to create images of discrete cellular locations. The corresponding average spectra for each ROI is plotted using Origin.

## Data availability

The research data associated with this paper will become available from the University of Strathclyde at the following link: https://doi.org/10.15129/3805140b-62f4-448b-b7ae-7ffd3c223a81.

## Author contributions

W. J. T., C. A. and A. A. L. performed the experiments. C. W. is responsible for maintaining cell cultures. W. J. T., L. T. W., A. W. W., N. C. O. T., K. F. and D. G. designed the project and analysed the results. W. J. T., N. C. O. T., K. F. and D. G. drafted the manuscript. N. C. O. T, K. F. and D. G. are responsible for funding.

## Conflicts of interest

There are no conflicts to declare.

## Supplementary Material

SC-013-D1SC06976D-s001
