# Supplementary material for: Stimulated Raman scattering microscopy with spectral phasor analysis: applications in assessing drug–cell interactions
Source: Chem Sci. 2022 Feb 25;13(12):3468–76. doi: 10.1039/d1sc06976d (PMC8943890; doi:10.1039/d1sc06976d)
Supplement: SC-013-D1SC06976D-s001 [file SC-013-D1SC06976D-s001.pdf]

Electronic Supplementary Material (ESI) for Chemical Science.
This journal is © The Royal Society of Chemistry 2022

Stimulated Raman scattering microscopy with spectral phasor analysis: applications in assessing drug-cell interactions

Electronic Supplementary Information

*William J. Tipping,[a] Liam T. Wilson,[b] Connie An,[‡a] Aristea A. Leventi,[a] Alastair W. Wark,[a] Corinna Wetherill,[a] Nicholas C. O. Tomkinson,[*b] Karen Faulds,[*a] and Duncan Graham[*a]*

[a.] *Centre for Molecular Nanometrology, WestCHEM, Department of Pure and Applied Chemistry, Technology and Innovation Centre, University of Strathclyde, 99 George Street, Glasgow G1 1RD, UK.*

[b.] *Department of Pure and Applied Chemistry, University of Strathclyde, 295 Cathedral Street, Glasgow G1 1XL, UK.*

‡Present address: *Department of Chemical and Biological Engineering, Princeton University, Princeton, NJ 08544, USA.*

Contents

Materials and Methods……………………………………………………………………………2

Figure S1-S10………………………………………………………………………………………3

Materials and Methods

Immunofluorescence staining

MCF-7, SK-BR-3 and MDA-MB-231 cells were plated on high precision glass coverslips (#1.5H Thickness, 22 × 22 mm, Thorlabs) in a 6-well plate in RPMI at a concentration of 1×10^6 cells per mL and incubated at 37 °C and 5% CO_2 for 24 h prior to treatment. The cells were washed with PBS (3 × 2 mL) and fixed using paraformaldehyde (4% in PBS, 15 min at rt). The cells were then washed with PBS (3 × 2 mL, 5 min each). The fixed cells were permeabilised using TritonX-100 (0.1% *v/v* in PBS, 10 min at rt). The cells were further washed with PBS (3 × 2 mL) before blocking with BSA (1% *v/v* in 0.1% PBS-Tween containing 22.5 mg mL^{-1} glycine) for 1 h. The cells were then incubated with the diluted antibody (ab16660) (1/200 dilution in 1% BSA in PBS-Tween) overnight at 4 °C. The cells were then washed in PBS (3 × 2 mL, 5 min each). Finally, the cells were incubated with the secondary antibody (ab150077 conjugated with AlexaFluor® 488) (1/200 dilution in 1% BSA) for 1 h at room temperature in the dark. The cells washed with PBS (2 × 2 mL, 5 min each) before counterstaining with DAPI (5 μM for 15 min). The coverslips were then affixed to glass microscope slides with a PBS boundary between the glass layers prior to imaging.

Fluorescence imaging

Fluorescence images were acquired using a Leica Microsystems SP8 confocal microscope equipped with a 63× NA 1.20 HC PL water immersion objective lens. DAPI staining was imaged using a 405 nm diode laser for excitation and a photon multiplier tube (PMT) detector set to acquire emitted wavelengths across the range 410-480 nm. Estrogen receptor-α (ERα) was imaged using a 488 nm argon-ion laser for excitation and a PMT detector set to acquire emitted wavelengths across the range 500-580 nm. Quantification of the mean fluorescence intensity per nucleus was performed on ImageJ using the DAPI image as a marker for the nuclear area, with subsequent quantification of the ERα signal per nucleus from a minimum of 150 cells from each cell population.

Raman spectroscopy

All Raman spectra were acquired on a Renishaw inVia Raman microscope equipped with a 532 nm Nd:YAG laser providing a maximum output of 50 mW and using a 1800 lines per mm grating. Prior to spectral acquisitions, the instrument was calibrated using the internal silicon standard at 520.5 cm^{-1}.

Raman imaging: MCF-7, SK-BR-3 and MDA-MB-231 cells were plated on glass-bottomed culture dishes (35 mm high, Ibidi) for imaging in the high wavenumber region, or cells were plated on glass-bottomed culture dishes (35 mm high, Ibidi) containing a Raman-grade CaF_2 disc (Crystran, UK) for imaging in the fingerprint region at a concentration of 5×10^5 cells per mL and incubated at 37 °C and 5% CO_2 for 24 h prior to treatment. Cells were treated with atorvastatin from a 50 mM stock solution in DMSO (or DMSO as a control) and incubated at 37 °C and 5% CO_2 for the indicated time. Prior to imaging, the dishes were aspirated and washed with PBS (2 × 2 mL), fixed with paraformaldehyde (4% in PBS, 15 min at room temperature). Cells were imaged in PBS. Raman maps were acquired using λ_{ex} = 532 nm with a Nikon 60×, N.A. 1.0 NIR Apo water immersion objective, 1 μm step size in x and y, 0.5 s or 1 s acquisition time, 50% laser power (*ca.* 18 mW) and a spectral centre of 1250 cm^{-1} or 2800 cm^{-1}. Three replicate maps of different cells were acquired from a single culture plate for each condition.

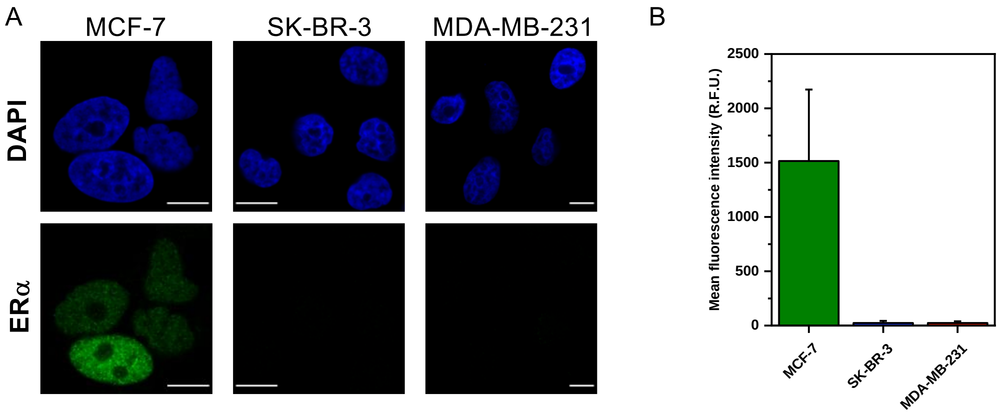

Figure S1 Characterisation of breast cancer cells for Estrogen Receptor-α (ERα) expression. **A** Determining ERα expression in breast cancer cell lines using immunofluorescence imaging. MCF-7, SK-BR-3 and MDA-MB-231 cells were stained with ERα antibody, secondary labelled with anti-rabbit AlexaFluor™ 488 and counter stained with DAPI nuclear stain. Scale bars: 10 µm. **B** Quantification of ERα expression in each cell line. The mean intensity of the ERα signal in the nucleus of each cell (>150 cells per condition) is plotted for each cell line. Error bars: +S.D.

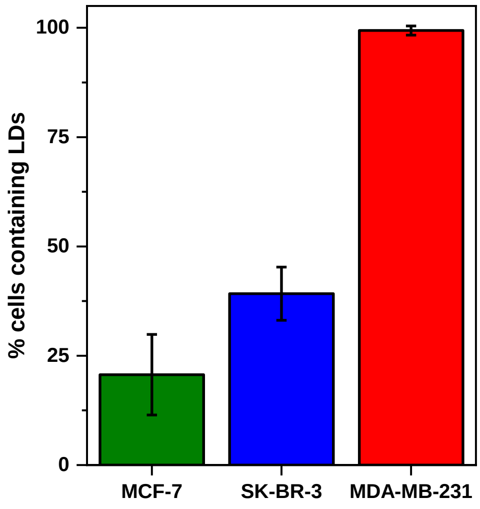

Figure S2 Analysis of the percentage of breast cancer cells containing lipid droplets. SRS image Z-stacks were acquired from live MCF-7, SK-BR-3 and MDA-MB-231 cells at 2851 cm^{-1} (CH_2, lipids) using 1 µm step in Z direction of the same cellular region presented in **Figure 1**. The data is plotted as the mean percentage of cells containing lipid droplets, error bars: ±S.D.

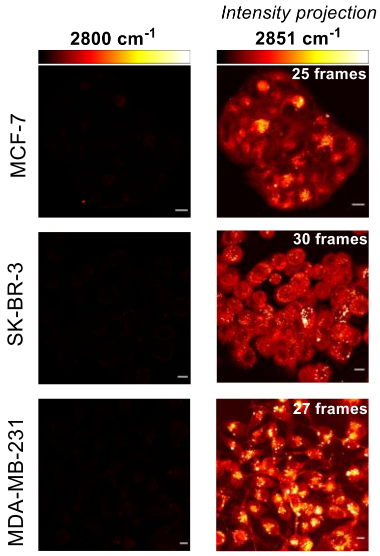

Figure S3 Off-resonance SRS imaging in breast cancer cells. SRS images were acquired from live MCF-7, SK-BR-3 and MDA-MB-231 cells at 2800 cm^{-1} (off-resonance; cell silent region) and compared to maximum intensity projections of the 2851 cm^{-1} (CH_2, lipids) from Z-stacks (1 µm step in Z direction) of the same cellular region. The images are presented in the red hot LUT scaled 0 – 2500 a.u. (to match the images acquired at 2851 cm^{-1} in **Figure 1**). Scale bars: 10 µm.

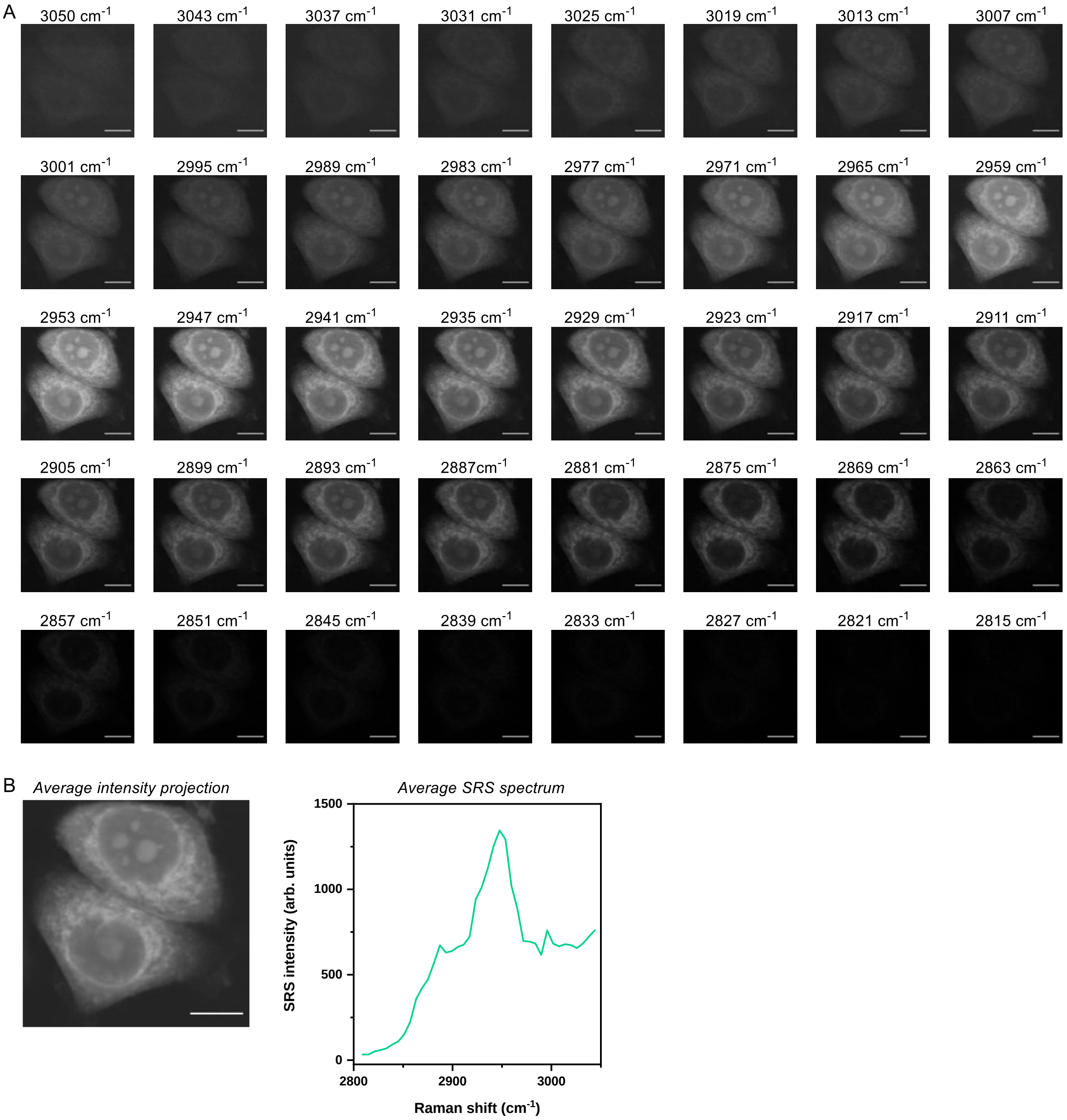

Figure S4 SRS imaging across the spectral range 2815-3050 cm^{-1} in SK-BR-3 cells. The wavelength of the pump beam was adjusted by approximately 0.4 nm between each image acquisition. The individual image frames were combined into a 3D image stack, from which the average intensity projection at each pixel could be determined, along with an average SRS spectrum across the stack. **A** Wavelength scanning SRS imaging of fixed SK-BR-3 cells. **B** Average intensity projection and average SRS spectrum from all pixels within the image stack. Scale bars: 10 μm.

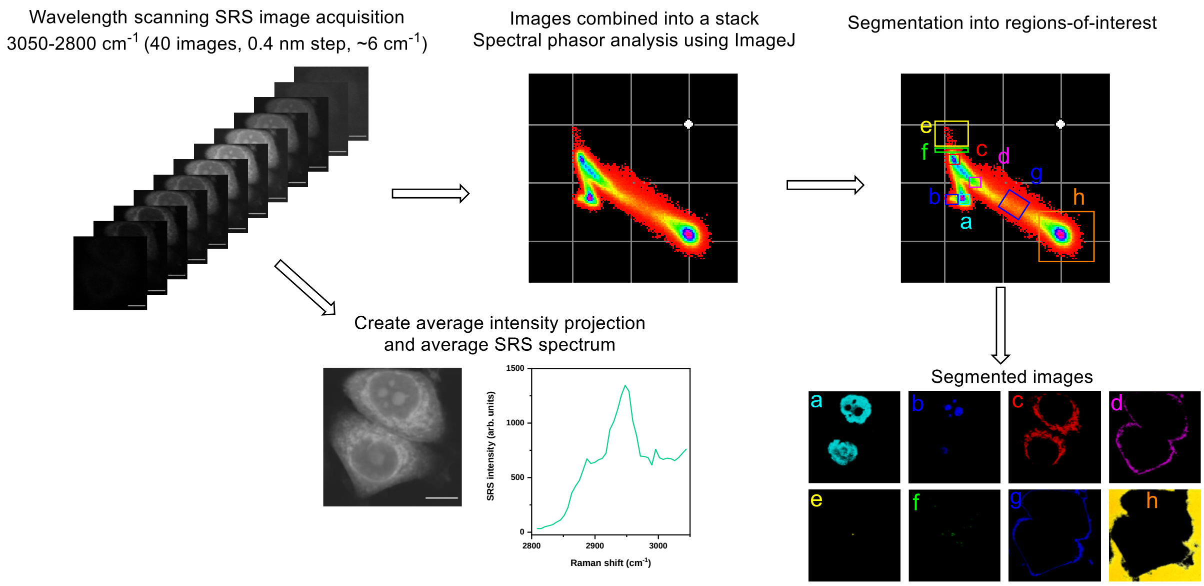

Figure S5 Data acquisition and analysis for SRS imaging and spectral phasor analysis. The spectral phasor plugin for ImageJ is available online and further details relating to the plugin can be found in D. Fu *et al*., *Anal. Chem.*, **2014**, *86*, 4115-4119.

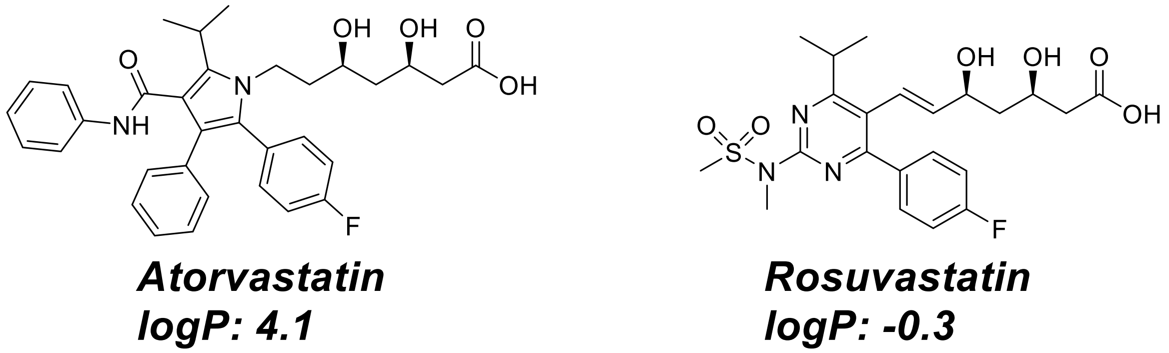

Figure S6 Chemical structures of the two stains investigated in this study. LogP values are reported in B. S. Wiggins *et al., Circulation*, **2016**, *134*, e468-e495.

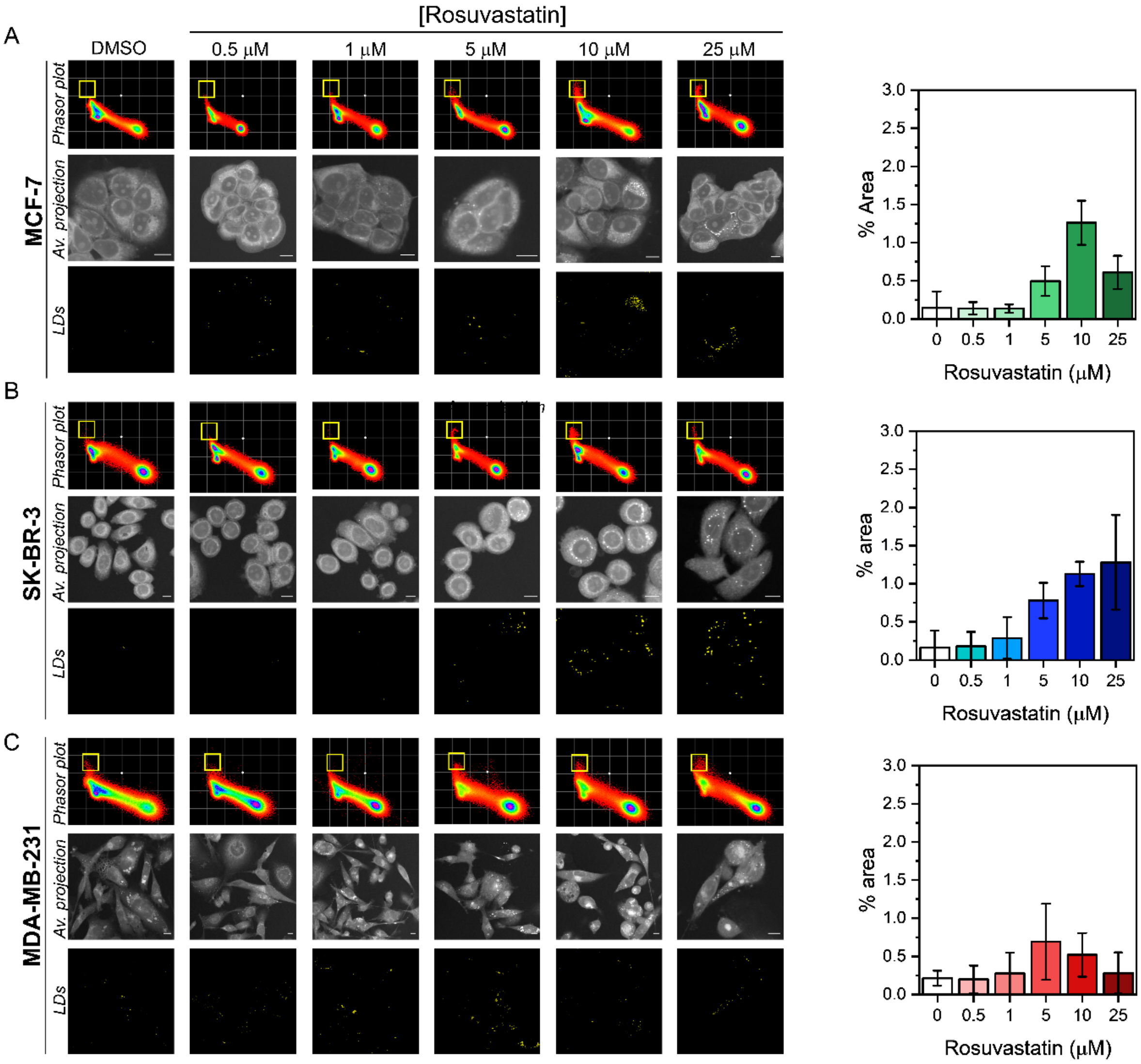

Figure S7 Investigating the effect of rosuvastatin in breast cancer cells using a spectral phasor approach. Spectral phasor plots were generated from **A** MCF-7, **B** SK-BR-3 and **C** MDA-MB-231 cells treated with rosuvastatin at the indicated concentrations for 48 h. An average intensity projection from the SRS spectral sweep is presented alongside the segmented phasor plot indicating lipid droplets (identified by the yellow ROI in the spectral phasor plot). Scale bars: 10 μm. The percentage area of lipid droplets in the segmented image is expressed as a function total cell area (determined from the average intensity projection). Data represent the mean area ± S.D.

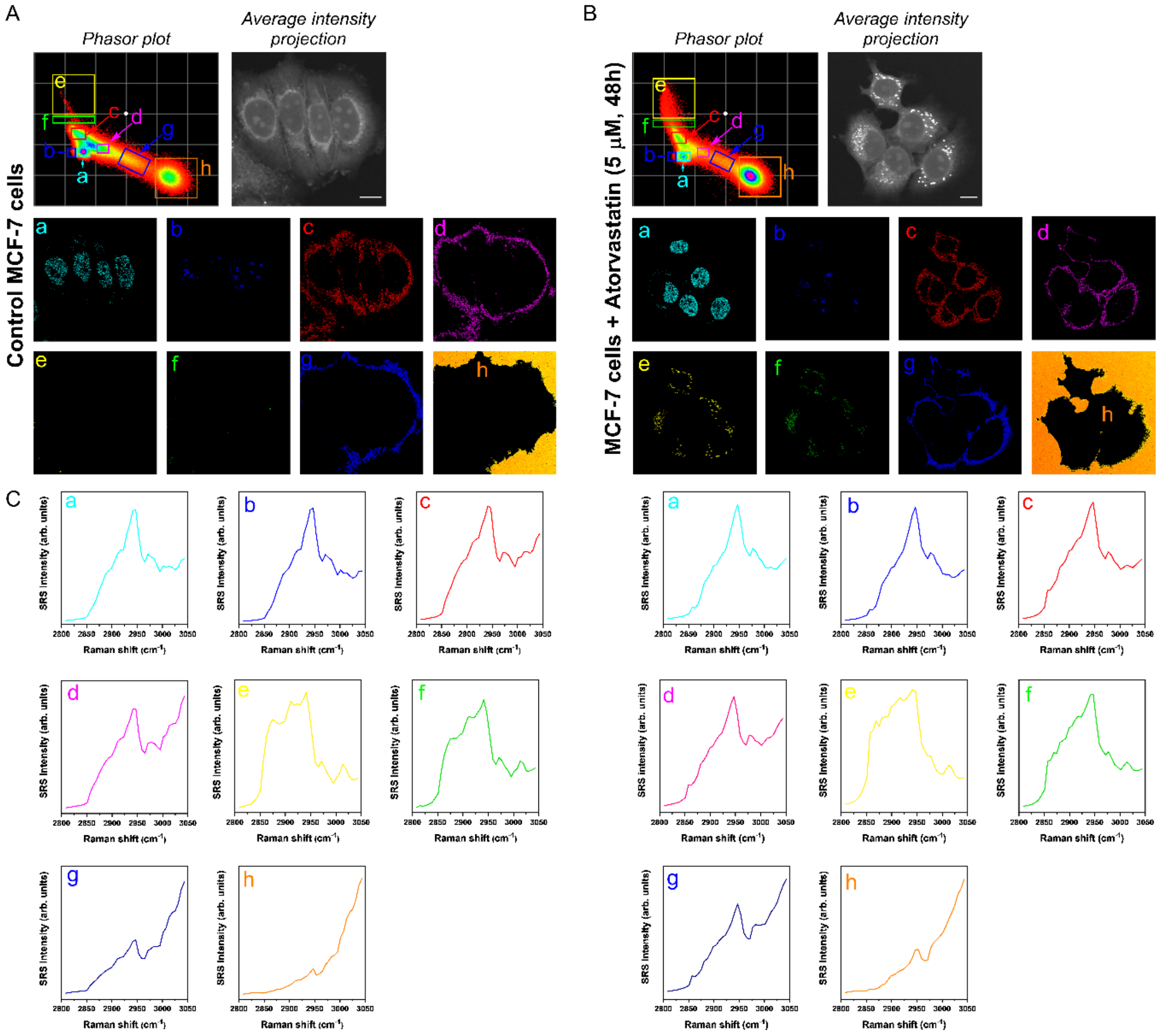

Figure S8 Analysis of MCF-7 cells using a spectral phasor method. SRS spectral sweeps were acquired across the range 2810-3050 cm^{-1} in a fixed cell population. Spectral phasor analysis of **A** control MCF-7 cells (0.01% DMSO, 48h) and **B** MCF-7 cells treated with atorvastatin (5 µM, 48h). An average intensity projection of the cell population is provided alongside the phasor plot, scale bar: 10 µm. Colour-coded images of eight segments (labelled a-h) of the phasor plots in each treatment condition with differing cellular locations are also included. **C** Corresponding average SRS spectra for the eight segmented images (labelled a-h) of the phasor plots identified in **A** and **B**.

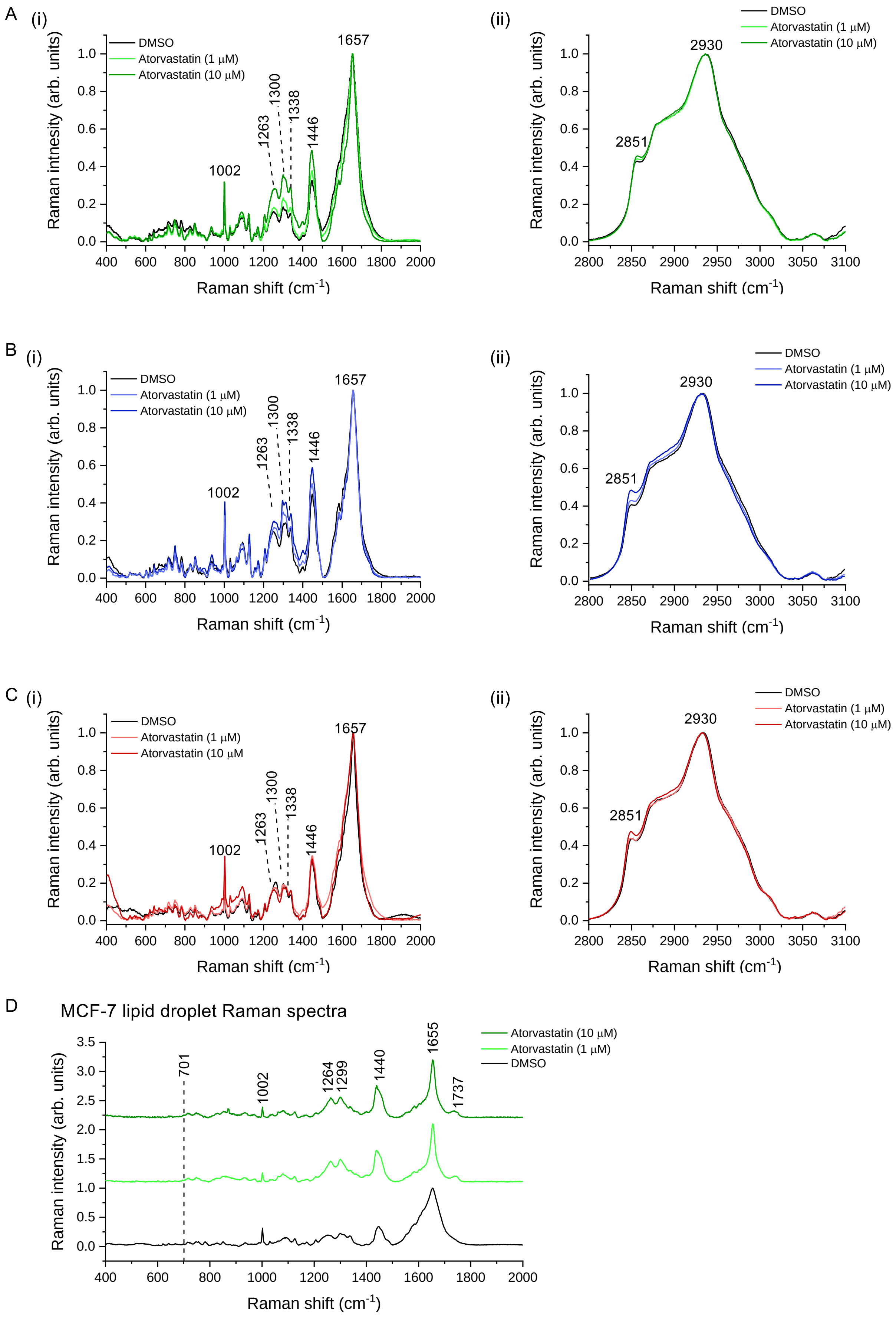
A (i)
DMSO
Atorvastatin (1 μM)
Atorvastatin (10 μM)
Raman intnesity (arb. units)
Raman shift (cm-1)
1002
1263
1300
1338
1446
1657
(ii)
2851
2930
Raman intensity (arb. units)
B (i)
Raman intensity (arb. units)
C (i)
Atorvastatin (10 μM
D
MCF-7 lipid droplet Raman spectra
701
1264
1299
1440
1655
1737

Figure S9 Raman spectral analysis of breast cancer cells exposed to atorvastatin. Breast cancer cells were treated with DMSO (control), atorvastatin (1 μM) or atorvastatin (10 μM) for 48 h in **A** MCF-7, **B** SK-BR-3 and **C** MDA-MB-231 cells. Individual cells were imaged using Raman spectroscopy and the average Raman spectrum is presented in the range (i) 400-2000 cm^{-1} or (ii) 2800-3100 cm^{-1}. Raman spectra are normalised to the intensity of the amide-I band at 1657 cm^{-1} in (i) and to the intensity of the CH_3 symmetric stretch at 2930 cm^{-1} in (ii). **D** Raman spectrum of lipid droplets/lipid-rich regions from MCF-7 cells. The annotation at 701 cm^{-1} (cholesterol ring vibration) is largely negligible, indicating the cholesterol content is low in the lipid droplets. Peak annotations in cm^{-1}. Raman spectra were acquired using 532 nm excitation with a 60× lens (18 mW) for (i) 1 s or (ii) 0.5 s using a 1 μm pixel size.

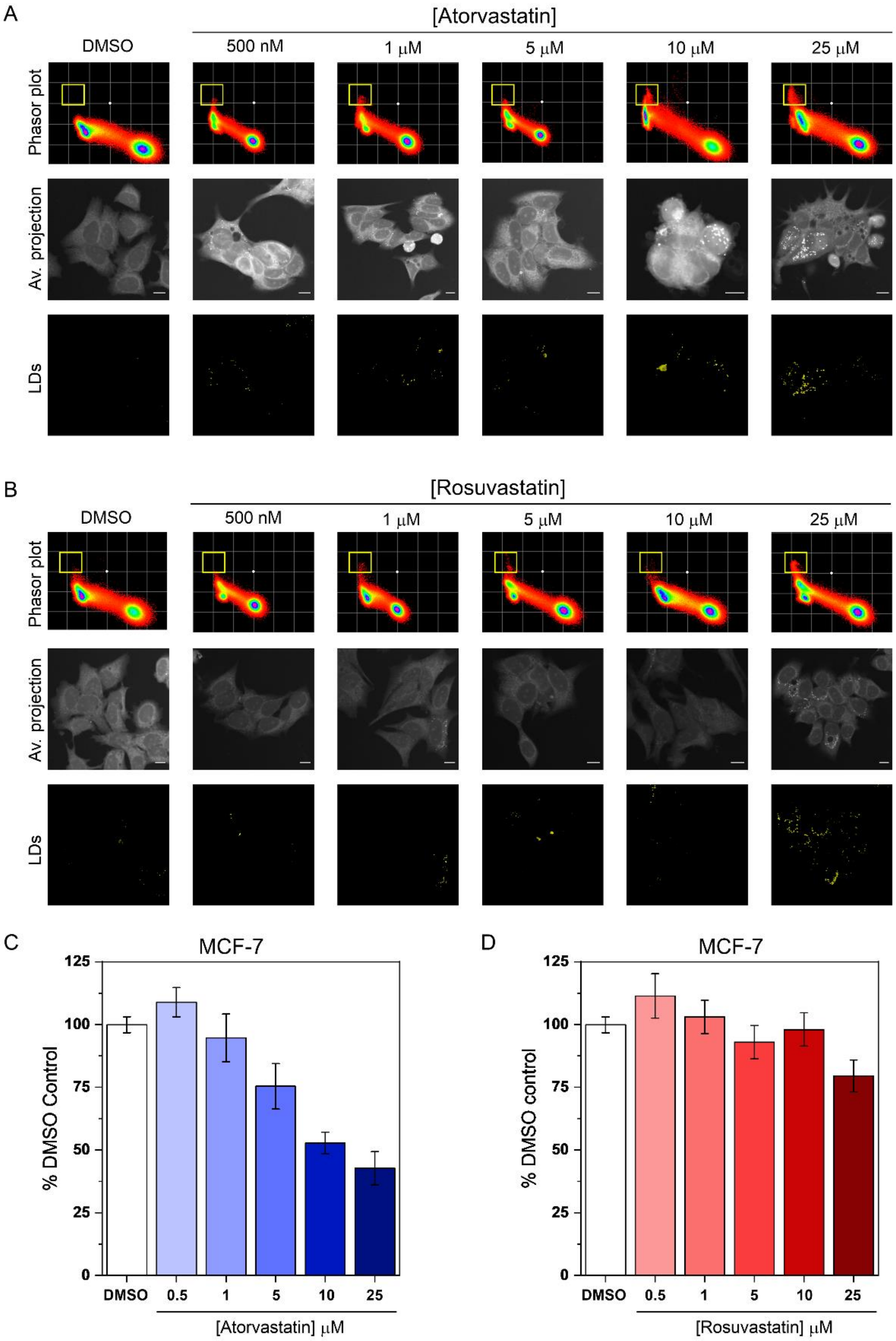

Figure S10 Investigating the effect of statin treatment in MCF-7 cells in serum-free media. Spectral phasor plots were generated from **A** MCF-7 cells treated with atorvastatin (0-25 μM, 48 h) or **B** MCF-7 cells treated with rosuvastatin (0-25 μM, 48 h) in serum-free RPMI media. An average intensity projection from the SRS spectral sweep is presented alongside the segmented phasor plot indicating lipid droplets (identified by the yellow ROI in the spectral phasor plot). Scale bars: 10 μm. **C, D** Cells were exposed to DMSO (control) or an increasing concentration of either atorvastatin (**C**) or rosuvastatin (**D**) in serum-free media for 48 h. The number of viable cells was determined using Trypan Blue staining and expressed as a mean% of the DMSO control with error bars: ±S.D.
